# Fine-Tuning Protein Fate: Mechanisms of E1, E2, and E3 Enzymes and Deubiquitinases in Cell Signaling

**DOI:** 10.3390/ijms27083404

**Published:** 2026-04-10

**Authors:** Yosup Kim, Eun-Kyung Kim, Ho Hee Jang

**Affiliations:** 1 Department of Health Sciences and Technology, Graduate School of Medicine, Gachon University, Incheon 21999, Republic of Korea; youandkys@gachon.ac.kr (Y.K.); ekkim@gachon.ac.kr (E.-K.K.); 2 Department of Biochemistry, College of Medicine, Gachon University, Incheon 21999, Republic of Korea

**Keywords:** ubiquitin, ubiquitination, proteasome, deubiquitinase, deubiquitination, ubiquitin–proteasome system, E1 enzymes, E2 enzymes, E3 enzymes

## Abstract

Ubiquitination is a reversible post-translational modification crucial for cellular homeostasis and protein degradation. It is orchestrated by a cascade of ubiquitin-activating enzymes (E1), conjugating enzymes (E2), and ligases (E3) that tag proteins with ubiquitin, and deubiquitinating enzymes (DUBs) that remove these tags. Through this tightly regulated ubiquitination/deubiquitination system, cells control protein turnover, localization, and activity, thereby governing processes ranging from cell cycle progression and DNA repair to immune and stress responses. Here, we review the structural and functional mechanisms of each class of enzymes in the ubiquitin–proteasome system, including E1, E2, E3, and DUBs, and highlight their roles in key signaling pathways and physiological processes. We further discuss how the dysregulation of these enzymes leads to diseases such as cancer, neurodegenerative disorders, and immune diseases, underlining the potential of targeting ubiquitination pathways for therapeutic intervention.

## 1. Introduction

The ubiquitin–proteasome system (UPS) is a central regulatory hub for protein quality control, ensuring proteostasis by the timely degradation or stabilization of intracellular proteins [1]. Its discovery revolutionized our understanding of protein turnover, which was once thought to be a nonspecific waste-disposal mechanism, and is now known to be a highly selective process for regulating virtually every cellular pathway [2]. Ubiquitin is a 76 amino-acid protein tag covalently attached to target proteins, marking them for a specific fate in the cell [3]. Ubiquitination occurs through a multi-enzyme cascade. The E1 ubiquitin-activating enzyme (E1), E2 ubiquitin-conjugating enzyme (E2), and E3 ubiquitin ligase (E3) act sequentially to attach ubiquitin to lysine (Lys) residues on substrates. These modifications can range from a single ubiquitin (monoubiquitination) to chains of ubiquitin (polyubiquitination) at various linkages, which encode different signals [4,5]. Dedicated deubiquitinases (DUBs) counterbalance this process by removing ubiquitin or editing ubiquitin chains, thereby allowing dynamic control of protein fate [6].

Ubiquitination affects virtually all aspects of cell biology. It regulates the cell cycle and mitosis, controls apoptotic cell death pathways, governs deoxyribonucleic acid (DNA) damage responses and repair, and fine-tunes immune and inflammatory signaling cascades, as well as autophagy and metabolic adaptations [7,8]. For example, oscillations in cyclin levels during the cell cycle are enforced by E3 ligases, such as the anaphase-promoting complex/cyclosome and SKP1-CUL1-F-box protein (SCF) complexes, which ubiquitinate cell cycle regulators at the appropriate time [9,10]. In immune signaling, K63-linked ubiquitin chains assembled by ligases, such as tumor necrosis factor (TNF) receptor-associated factor 6 (TRAF6) or cellular inhibitor of apoptosis protein-1, serve as scaffolds to activate nuclear factor kappa-light-chain-enhancer of activated B cells (NF-κB) and other pathways, whereas DUBs, such as A20, remove these chains to terminate signaling [11]. Given the breadth of the processes governed by the UPS, it is not surprising that its dysregulation is associated with numerous pathologies. Aberrant or accumulated ubiquitinated proteins are linked to neurodegenerative disorders, whereas excessive degradation of tumor suppressors or stabilization of oncoproteins drives many cancers [12,13]. Malfunctions of specific UPS components underlie certain inherited diseases and immune disorders [14,15]. In the following sections, we examine in detail the four major classes of enzymes that constitute the UPS—E1, E2, E3, and DUBs—describing their structural and mechanistic features, their roles in cellular signaling pathways, and examples of how their dysfunction contributes to disease.

## 2. The Enzymatic Architecture of the Ubiquitin System

**Figure 1:**
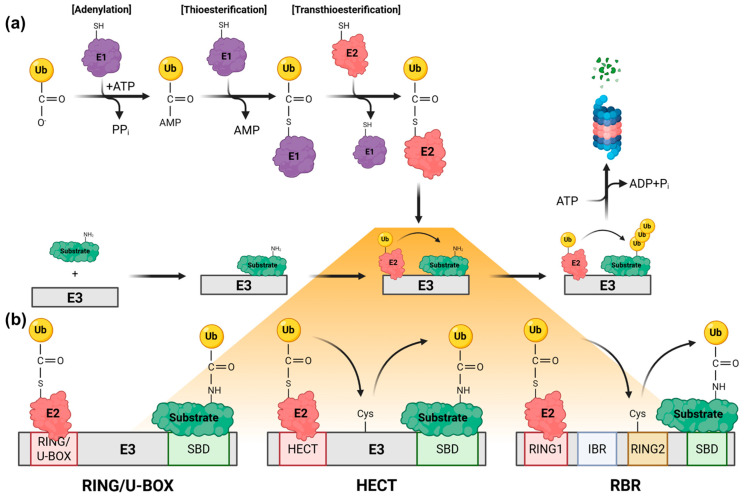
Overview of the ubiquitination cascade and major E3 ligases. (**a**) The ubiquitin-proteasome system. Ubiquitination proceeds through the sequential actions of E1 (activating), E2 (conjugating), and E3 (ligase) enzymes. First, E1 enzymes activate ubiquitin in an ATP-dependent manner (adenylation/thioesterification) and transfer it to the active site cysteine of E2 enzymes (transthioesterification). Subsequently, E3 enzymes recognize the substrate and facilitate the attachment of ubiquitin to a lysine (Lys) residue on the substrate. The formation of Lys48 (K48)-linked polyubiquitin chains via repeated cycles targets the substrate to the 26S proteasome for degradation. (**b**) Classification of E3 Ligases. E3 ligases are categorized into three major families based on their mechanism of ubiquitin transfer. Really interesting new gene (RING)/U-BOX E3 enzymes function as a scaffold to bring E2 enzymes and the substrate into proximity, facilitating the direct transfer of ubiquitin from the E2 enzyme to the substrate. Homologous to E6-associated protein (E6-AP) C-terminus [HECT] E3 enzymes accept ubiquitin from E2 enzymes to form a covalent E3–ubiquitin thioester intermediate on their own active site cysteine (Cys) residue before transferring it to the substrate. RING-Between-RING (RBR) E3 enzymes employ a hybrid mechanism. The RING1 domain binds E2–ubiquitin, and ubiquitin is transferred to a cysteine residue on the RING2 domain to form an intermediate prior to its final transfer to the substrate. Figure was created in BioRender. https://BioRender.com/82ktjyi (access on 4 April 2026).

The conjugation of ubiquitin to a substrate is a tightly regulated adenosine triphosphate (ATP)-dependent process orchestrated by a sequential enzymatic cascade (Figure 1) [16]. This modular system allows for an immense combinatorial diversity of outcomes, enabling ubiquitin to regulate processes such as cell division and the immune response.

### 2.1. The Gatekeepers: Ubiquitin-Activating Enzymes (E1)

E1 enzymes initiate the ubiquitination cascade via the ATP-dependent activation of ubiquitin. E1 enzymes are large (~110 kDa) molecules with multiple domains, including an adenylation domain that binds ATP–ubiquitin, a Cys-containing catalytic domain, and a ubiquitin-fold domain that recruits specific E2s [17]. The activation mechanism of E1 enzymes involves a two-step reaction.

1. The E1 enzyme adenylates the C-terminal glycine (Gly) of ubiquitin using ATP, forming a high-energy ubiquitin–AMP intermediate.
2. Ubiquitin is transferred to the catalytic Cys residue on the E1 enzyme to form a thioester-linked E1–ubiquitin conjugate. The activated ubiquitin is then handed off to an E2-conjugating enzyme [18].

In humans, there are two E1 enzymes, ubiquitin-activating enzyme E1 (UBE1, encoded by the *UBA1* gene) and UBE1-like protein 2 (UBE1L2), which are dedicated to ubiquitin [19]. UBE1 is the predominant E1 enzyme that charges ubiquitin for transfer to all downstream E2 and E3 enzymes, while UBE1L2 is an E1 enzyme with unique substrate specificity. Notably, UBE1L2 has a unique C-terminal ubiquitin-fold domain that preferentially recruits the cognate E2 enzyme Ubiquitin-conjugating enzyme E2 Z. UBE1L2 can activate ubiquitin and the ubiquitin-like (UBL) protein, human leukocyte antigen-F adjacent transcript 10 (FAT10), effectively operating a distinct ubiquitin/FAT10 conjugation pathway in parallel with UBE1. This dual specificity of UBE1L2 highlights that E1 enzymes can have specialized roles beyond a simple backup. UBE1L2 directs ubiquitin to a subset of E2–E3 enzyme pathways and is inducible under certain cellular stress conditions, whereas UBE1 handles the bulk of the constitutive ubiquitination reactions [20].

Although E1 enzymes are located at the beginning of the UPS cascade, and are comparatively few in number, their appropriate functioning is vital. Mutations in the *UBA1* gene can lead to serious diseases; for example, X-linked infantile spinal muscular atrophy is caused by loss-of-function variants of *UBA1*, resulting in the degeneration of motor neurons in affected male infants [21]. This provides strong evidence that even partial impairment of ubiquitin activation disrupts proteostasis in specific tissues (in this case, neuromuscular units). Conversely, E1 enzyme inhibitors, such as TAK-243, have been explored as potential anticancer agents that suppress ubiquitination and cause the accumulation of proteins that are toxic to cancer cells [22]. However, such approaches are associated with high toxicity, given the ubiquitous roles of ubiquitin.

### 2.2. The Architects of Chain Topology: Ubiquitin-Conjugating Enzymes (E2)

E2 enzymes are ubiquitin-conjugating enzyme E2 (UBE2) encoded by approximately 35–40 genes in the human genome, each characterized by a conserved ~150-amino-acid ubiquitin-conjugating (UBC) domain that contains a catalytic Cys residue positioned within a shallow active-site groove. This UBC domain has a characteristic β-sheet structure and flanking helices that provide an interface for E3 binding [23,24]. Activated ubiquitin is transferred from E1 to E2 enzymes via a transthioesterification reaction, in which ubiquitin is accepted by the catalytic cysteine residue of the E2 enzyme [25].

E2 enzymes act as linchpins that define ubiquitin modification. The E2–E3 enzyme pair determines whether a substrate is mono-ubiquitinated or polyubiquitinated [26]. If polyubiquitination occurs, the same E2–E3 enzyme combination specifies the linkage type of the ubiquitin chain. Different E2 enzymes exhibit preferences for certain ubiquitin–lysine linkages; for example, the E2 enzyme UBC13 forms K63-linked chains of rhomboid 5 homolog 2, which is important in DNA damage and NF-κB signaling, by working with cofactor ubiquitin-conjugating enzyme E2 variant 1 proteins, whereas UBE2C and UBE2S primarily assemble K11-linked chains during cell cycle progression [27,28]. Even the same E2 enzyme can catalyze different chain linkages, depending on its E3 enzyme partner. UBE2D3 predominantly catalyzes K48-linked polyubiquitin when paired with the HECT-type ubiquitin ligase E6-AP, but switches to K6-linked chains when interacting with the breast cancer type 1 susceptibility protein (BRCA1)–BRCA1-associated RING domain 1 E3 complex [29,30]. This versatility means that E2 enzymes are not merely passive carriers that transfer ubiquitin, but instead actively shape the structure and functional characteristics of ubiquitin signaling. Overall, E2 enzymes provide a crucial midpoint of regulation in the UPS, serving as hubs where signals from E1 enzymes (ubiquitin activation) are translated in concert with E3 enzymes into specific ubiquitin modifications of targets [31].

### 2.3. The Specificity Factors: Ubiquitin Ligases (E3)

E3 ubiquitin ligases are the key specificity determinants of the UPS and are responsible for recognizing target substrates and facilitating or catalyzing the transfer of ubiquitin from E2 enzymes to residues (such as serine, threonine, Cys, and the N-terminus) within substrate proteins [32]. The human genome encodes 600–700 E3 enzymes, reflecting a diverse array of protein substrates and cellular processes regulated by ubiquitin [33,34]. E3 enzymes are broadly classified into three major families based on their catalytic mechanisms and structural domains: RING, HECT, and RBR, with U-box-type ligases considered a sub-type of RING E3s (Table 1) [35,36,37].

#### 2.3.1. RING-Type E3 Ligases and U-Box E3 Ligases

RING-type E3 ligases constitute the largest E3 ligase family, accounting for more than 90% of all human E3 ubiquitin ligases [38]. These ligases have a RING finger domain that coordinates two zinc ions (except for the zinc-independent U-box) and binds directly to the E2–ubiquitin conjugate [39]. Crucially, RING E3 enzymes act as scaffolds and catalytic activators, positioning E2–ubiquitin and stabilizing a closed conformation that promotes the transfer of ubiquitin from E2 to the substrate without forming an E3–ubiquitin intermediate [35]. RING E3 enzymes can function as simple units (monomeric or homodimeric), such as MDM2 and CHIP, or as multi-subunit complexes [40,41]. A prominent example of the latter is the Cullin-RING Ligase (CRL) family, similar to the SCF complex, which uses a Cullin scaffold, adaptor proteins, receptor proteins, and RING proteins to bring substrate receptors and RING-E2 together. These modular CRL complexes allow for interchangeable substrate receptors, dramatically expanding the substrate specificity of RING E3 enzymes [42,43,44]. Overall, RING ligases typically promote polyubiquitination linked to proteasomal degradation, although some ligases (e.g., TRAF6 and tripartite Motif proteins) assemble K63 chains for signaling [45,46,47].

The U-box E3 ligase family is a small group of enzymes that are structurally related to RING ligases, but lack coordinated zinc ions. Instead, the U-box domain is stabilized by a network of hydrogen bonds and salt bridges that maintain a RING-like fold capable of binding to an E2–ubiquitin conjugate and promoting direct ubiquitin transfer [48,49]. Several U-box proteins function in protein quality control pathways, in cooperation with molecular chaperones. A well-known example is CHIP, also known as stress-induced-phosphoprotein 1 homology and U-box containing protein 1, which associates with heat shock protein 70 (Hsp70) and Hsp90 to ubiquitinate misfolded client proteins, targeting them for proteasomal degradation, thereby protecting cells from proteotoxic stress [50].

Despite mechanistic differences, all E3 ligases play a common role in substrate selection in the UPS. They typically recognize specific sequence motifs or post-translational modifications in target proteins (called degrons). Some E3s have dedicated adaptor or scaffold subunits that expand their range of substrates [51]. Given their central role in determining substrate specificity, E3 ligases represent critical checkpoints in the ubiquitin system, and their dysregulation is frequently linked to human diseases.

#### 2.3.2. HECT-Type E3 Ligases

The HECT-type E3 ligases are a family of approximately 28 human enzymes that operate via a two-step catalytic mechanism. These enzymes contain a C-terminal HECT domain with an essential catalytic Cys residue. First, ubiquitin from E2 is transferred to Cys, forming a transient E3–ubiquitin thioester intermediate. Next, ubiquitin is ligated to the E3 substrate. This catalytic mechanism allows HECT E3 ligases to directly influence the initiation and elongation of ubiquitin chains. For instance, some HECT ligases, particularly members of the NEDD4 family, have been reported to preferentially assemble K63-linked chains, although the chain types can vary depending on the associated E2 enzyme and substrate context [52,53,54].

HECT-type E3 ligases have modular N-terminal domains (such as tryptophan–tryptophan [WW] or WW–glutamate domains) for substrate or regulator binding, defining the NEDD4 subfamily (nine members with C2 and WW domains), the HERC subfamily (six large enzymes with regulator of chromosome condensation 1-like domains), and other HECTs (approximately 13 members with diverse domains) [55,56]. A well-known example is E6-AP, which ubiquitinates p53 in the presence of human papillomavirus type 16 E6 oncoprotein and acts as an adaptor to recruit p53 to E6-AP [57]. Loss of E6-AP function causes a neurological disorder, Angelman syndrome, which highlights the physiological importance of HECT E3 ligases [58].

#### 2.3.3. RBR-Type E3 Ligases (RING/HECT Hybrid)

RBR-type E3 ligases constitute a distinct E3 family that includes Parkin, HOIP, and Heme-oxidized IRP2 ubiquitin ligase-1 (HOIL-1, a component of the linear ubiquitin chain assembly complex [LUBAC]) [59]. These ligases possess a characteristic tripartite domain architecture composed of RING1–in-between-RING (IBR)–RING2 domains. RBR ligases employ a hybrid RING-HECT catalytic mechanism. The RING1 domain binds to E2–ubiquitin like a RING ligase; however, the RING2 (also termed Rcat) domain accepts ubiquitin onto its catalytic cysteine to form a transient thioester-linked E3–ubiquitin intermediate before transferring it to the substrate. Thus, RBR ligases utilize a mechanism that combines the features of both the RING and HECT families [60].

Parkin is a well-characterized RBR ligase that ubiquitinates outer mitochondrial membrane proteins during mitophagy and is responsible for the clearance of damaged mitochondria. Loss-of-function mutations in parkin are the major causes of autosomal recessive familial Parkinson’s disease [61,62]. Another important example is the LUBAC, which is composed of HOIP, HOIL-1, and SHANK-associated RH domain-interacting protein. Within this complex, HOIP serves as a catalytic subunit that assembles linear (M1-linked) ubiquitin chains, which are critical for NF-κB signaling, whereas HOIL-1 primarily plays a regulatory role and can catalyze atypical ester-linked ubiquitination [63,64].

### 2.4. The Eraser and Editor: Deubiquitinating Enzymes (DUBs)

DUBs are proteases that specifically cleave ubiquitin from substrates or polyubiquitin chains by hydrolyzing the isopeptide bond between the C-terminal Gly76 of ubiquitin and the ε-amino group of Lys residues on substrate proteins or within ubiquitin chains (and peptide bonds in linear M1-linked chains) [65,66]. The human genome encodes approximately 100 DUBs that perform several essential functions in the UPS [67]. Physiologically, DUBs are indispensable regulators in signaling pathways. They provide a means to rapidly turn off a ubiquitin-dependent signal or to rescue a protein from degradation. It is important to note that UBL proteins, such as small ubiquitin-like modifier (SUMO) and NEDD8, are processed by distinct dedicated protease systems (e.g., SUMO-specific proteases for SUMO and NEDD8-specific Protease 1 for NEDD8), thereby preserving the specificity of the ubiquitin versus UBL signaling pathways [68,69,70].

1. Processing of newly synthesized ubiquitin precursors

Ubiquitin is synthesized either as a head-to-tail polyubiquitin precursor encoded by the *Ubb* and *Ubc* genes or as ribosomal fusion proteins encoded by *Uba52* and *Rps27a*, and must be proteolytically processed to generate free ubiquitin monomers [71].

2. Ubiquitin recycling

DUBs remove ubiquitin from substrates targeted by the proteasome and ubiquitin conjugates that would otherwise sequester ubiquitin, thereby maintaining the intracellular pool of free ubiquitin [6,72].

3. Editing and reversal of ubiquitin signals

DUBs edit, remodel, or completely remove ubiquitin chains to regulate protein stability, localization, and signaling [73].

DUBs are structurally classified into seven families. Six families (ubiquitin specific protease [USP], ubiquitin carboxy-terminal hydrolase [UCH], ovarian tumor protease [OTU], Machado–Joseph disease proteases [MJD/Josephin], motif interacting with ubiquitin-containing novel DUB family [MINDY], and zinc finger-containing ubiquitin peptidase [ZUFSP]) are cysteine proteases, whereas the Jun activation domain-binding protein 1/Mpr1-Pad1-N-terminal/MOV34 metalloenzyme [JAMM/MPN^+^] family comprises zinc-dependent metalloproteases (Table 2) [74,75].

#### 2.4.1. USP

The largest DUB family comprises approximately 56 members [76]. USPs are characterized by a conserved catalytic core domain and diverse regulatory regions that enable broad substrate specificity and context-dependent functions [77]. Prominent examples include USP7, which deubiquitinates and stabilizes the E3 ligase MDM2, thereby promoting degradation of the tumor suppressor p53 [78]. Another example is USP9X, which deubiquitinates the anti-apoptotic protein myeloid cell leukemia 1 to prevent proteasomal degradation [79].

#### 2.4.2. UCH

A small family of cysteine proteases (four members in humans: UCH-L1, UCH-L3, UCH-L5, and BAP1) generally possesses a compact catalytic domain of ~230 amino acids. A defining structural feature is the active-site crossover loop, which restricts access to the catalytic center and limits the ability of the minimal catalytic domain to process bulky protein substrates. Consequently, many UCH enzymes are particularly efficient in processing ubiquitin precursors or in removing ubiquitin from small adducts (such as short peptides or small chemical groups) [80]. UCH-L1 maintains the pool of free ubiquitin in cells [81]. UCH-L5 associates with the proteasome and participates in ubiquitin chain trimming, whereas BAP1 regulates chromatin regulation, including the deubiquitination of histone H2A [82,83].

#### 2.4.3. OTU

OTU family comprises 16 members in humans [84]. They are cysteine proteases distinguished by their remarkable linkage specificity, a defining feature that distinguishes them from many other DUB families [85]. For instance, OTUB1 preferentially cleaves K48-linked ubiquitin chains and exerts a non-catalytic inhibitory effect by binding to ubiquitin-charged E2 enzymes, thereby suppressing ubiquitination independent of its protease activity [86]. Many OTU family members function as critical negative regulators of immune and inflammatory signaling pathways. OTULIN is specific for linear (M1-linked) ubiquitin chains [87]. A20 removes K63-linked chains and promotes K48-linked ubiquitination through its zinc finger domains, facilitating the termination of NF-κB signaling [88]. Similarly, OTU Deubiquitinase 7B regulates NF-κB activation by modulating K63-linked ubiquitin chains [89].

#### 2.4.4. MJD

MJDs are a small family of cysteine protease-deubiquitinating enzymes, including ataxin-3, ataxin-3-like protein, JOSD1, and JOSD2 [90]. Ataxin-3 is mutated in Machado–Joseph disease (spinocerebellar ataxia type 3), a neurodegenerative disorder caused by polyglutamine expansion [91]. MJDs cleave ubiquitin chains and are involved in the protein quality control pathways. Ataxin-3 primarily functions as a polyubiquitin chain-editing enzyme, preferentially trimming longer ubiquitin chains on selected substrates. Through interactions with components of the proteostatic network (e.g., p97), ataxin-3 has been proposed to regulate substrate processing and degradation. Pathogenic expansion of polyglutamine disrupts these functions and contributes to neurodegeneration [90].

#### 2.4.5. MINDY

MINDY proteins represent a recently identified family of cysteine-based deubiquitinating enzymes (comprising five members in humans, MINDY1–4 and MINDY4B) with pronounced specificity for K48-linked polyubiquitin chains [6]. The well-characterized members MINDY1 and MINDY2 exist in an autoinhibited state, which is relieved only when sufficiently long K48-linked chains simultaneously engage multiple distinct ubiquitin-binding sites within the catalytic domain. This substrate-assisted activation induces a conformational change, ensuring strict specificity for cleaving long polyubiquitin chains, while displaying reduced activity toward short K48-linked chains. Mechanistically, MINDY enzymes function as molecular rulers that sense chain length. They can exhibit endo-DUB activity on longer K48-linked chains, enabling rapid chain shortening, but increasingly shifting toward exo-type trimming as the chains become shorter [92].

#### 2.4.6. ZUFSP

ZUFSP, also known as ZUP1, is an atypical cysteine protease-type deubiquitinase that is structurally and evolutionarily distinct from other cysteine DUB families. ZUFSP exhibits a strong preference for long K63-linked polyubiquitin chains and plays a critical role in DNA damage response [93]. It recognizes polyubiquitin chains through multiple ubiquitin-binding modules that allow discrimination based on chain length and topology. At sites of DNA damage, ZUFSP edits or remodels K63-linked ubiquitin chains, thereby modulating the ubiquitin-dependent assembly of DNA repair signaling complexes. Functionally, ZUFSP acts as a ubiquitin editor that modulates signaling ubiquitin scaffolds, rather than simply recycling ubiquitin [94].

#### 2.4.7. JAMM

The JAMM/MPN+ family comprises approximately 14 Zn^2+^-dependent metalloproteases, many of which function as DUBs. These proteins utilize a catalytic zinc ion instead of an active-site cysteine. JAMM proteins frequently function as integral components of large multi-protein complexes [95].

A prominent example is regulatory particle non-ATPase 11 (RPN11), a subunit of the 19S proteasome regulatory protein. RPN11 removes polyubiquitin chains en bloc from substrates in a degradation-coupled manner that is closely linked to substrate engagement and translocation into the proteasome, thereby enabling efficient proteolysis and ubiquitin recycling [16,96]. CSN5 serves as the catalytic subunit of the COP9 signalosome. Structurally, the JAMM metalloprotease CSN5, primarily acts as a deneddylase to remove NEDD8 from Cullin-RING ligases [97]. Another important member, BRCC36, functions within the BRCA1-A complex, specifically removing K63-linked ubiquitin chains at sites of DNA damage to regulate DNA repair signaling [98]. Other members are AMSH, a K63-linkage-specific DUB that regulates endosomal sorting within the endosomal sorting complexes required for transport pathway [99].

Despite the structural diversity across families, all DUBs share the fundamental ability to recognize ubiquitin and hydrolyze isopeptides or peptide bonds involving the C-terminus of ubiquitin [100,101]. Many DUBs are highly regulated to ensure that deubiquitination is coordinated with cellular needs. DUBs often exist in an auto-inhibited state or require binding partners and post-translational modifications to achieve full activity [102,103]. For instance, USP1 exhibits catalytic activity only when complexed with USP1-associated factor 1, and USP7 utilizes UBL domains to allosterically modulate its catalytic cleft upon ubiquitin binding [104,105].

## 3. Pathological Implications of UPS Dysregulation

The balance between ubiquitination and deubiquitination is critical for health, and its disruption has been recognized in a wide range of diseases. Dysregulation of E1, E2, E3, or DUB functions contributes to major diseases. Especially, among the UPS components, E3 ligases are the most frequently implicated in pathology because of their pivotal role as specificity determinants. Through regulated interactions, E3 enzymes act as the executing step in signaling pathways, determining when to degrade a protein or alter its activity. Consequently, aberrations in E3 function are common drivers of disease [106]. For instance, overexpression of MDM2, an E3 ubiquitin ligase, leads to excessive degradation of the tumor suppressor p53 in many cancers, whereas loss-of-function mutations in Fbw7 stabilize growth-promoting oncoproteins [107,108]. Beyond malignancy, E3 mutations underlie hereditary disorders such as Parkin-associated Parkinson’s disease and HOIL-1-linked immune syndromes [47,109]. Given their central role, E3 ligases have emerged as attractive targets for drug discovery efforts aimed at restoring normal protein homeostasis.

### 3.1. Cancer

Many cancers hijack the UPS to accelerate the degradation of tumor suppressors or stabilize oncogenes. For instance, overexpression of the E3 ligase MDM2 leads to excessive ubiquitination and degradation of p53, thereby inhibiting crucial tumor suppressor pathways [110].

Mutations that inactivate E3 enzymes responsible for oncoprotein degradation, may have a similar effect. The F-box protein FBXW7, a substrate receptor within the SCF E3 ubiquitin ligase complex, usually promotes the degradation of oncogenic regulators, including cyclin E, c-Myc, and Notch. F-Box and WD repeat domain containing 7 (FBXW7) is frequently mutated in colon, breast, and liver cancers, leading to the stabilization and accumulation of these substrates [111]. c-Myc, a regulator of cellular growth, is tightly controlled by SCF–FBXW7, which recognizes phosphorylated c-Myc via a phosphodegron and targets it for proteasomal degradation. FBXW7 mutations disrupt this recognition, leading to aberrant c-Myc accumulation and tumorigenesis [112,113]. Notably, a recently identified SCF adaptor, F-box and leucine-rich repeat protein 8, appears to regulate a distinct pool of c-Myc, supporting a multi-layered E3-ligase control architecture over c-Myc proteostasis [114].

DUBs also play a role in cancer progression. USP7 stabilizes MDM2 and murine double minute X, thereby promoting the inactivation of p53. High USP7 activity in tumors is associated with poor outcomes, and USP7 inhibitors are being explored to reactivate p53-mediated tumor suppression [78].

CYLD, another DUB, is a well-known tumor suppressor. This protein removes activating K63-linked ubiquitin chains from proteins in the NF-κB and Wingless-related integration site signaling pathway. Mutation or silencing of CYLD leads to active pro-growth and pro-survival signaling [115,116].

Regulators of the UPS (E1, E2, and E3 enzymes; DUBs; and proteasomes) are frequently dysregulated in cancers, and several are being targeted for drug development [117]. The success of proteasome inhibitors in multiple myeloma and ongoing trials of ligase inhibitors, such as MDM2 antagonists, underscore the centrality of the UPS in oncogenic processes [118].

### 3.2. Neurodegenerative Disorders

Protein homeostasis is critical in neurons, and UPS impairment is a common feature of neurodegenerative diseases. A prominent example is Parkinson’s disease (PD), in which early onset is frequently driven by mutations in PTEN-induced kinase 1 (PINK1) or Parkin [119]. Parkin is an E3 ligase activated in the PINK1-dependent mitophagy pathway, where it ubiquitinates damaged mitochondrial proteins and triggers selective mitochondrial clearance. Loss of this activity results in the accumulation of dysfunctional mitochondria, increased oxidative stress, and ultimately, neuronal death [120,121].

Likewise, mutations in other UPS components implicated in mitochondrial quality control, such as the E3 ligase F-Box Protein 7 and DUB USP30, which counteract Parkin-mediated ubiquitination, have also been linked to PD, collectively underscoring the central role of the UPS in mitophagy and neuronal survival [122,123].

In Alzheimer’s disease (AD), toxic protein aggregates (like Aβ and tau) may overwhelm or evade the UPS. Impaired proteasome activity and changes in E3 enzyme and DUB expression have been observed in the brains of patients [124]. For example, the levels of the U-box E3 ligase CHIP, which promotes the ubiquitination and clearance of misfolded and hyperphosphorylated tau, and the neuron-enriched DUB UCH-L1 have been reported to be altered in patients with AD, consistent with impaired ubiquitin-dependent tau quality control [124,125]. Reduced CHIP activity can facilitate tau accumulation and contribute to neurofibrillary tangle formation [126].

Furthermore, proteasome system dysfunction has emerged as a pathogenic driver of neurodegeneration, as exemplified by *UBQLN2* mutations in amyotrophic lateral sclerosis and by aberrant DUB engagement that impairs proteasome function in the P0 S63del model of Charcot–Marie–Tooth disease type 1B [127,128]. Overall, UPS dysfunction, whether because of an overwhelming number of proteasomes, mutant ligases, DUBs, or ubiquitin mutations, contributes to the accumulation of neurotoxic proteins [129]. Enhancing certain UPS activities and inhibiting harmful ones are currently being investigated as neuroprotective strategies.

### 3.3. Immune and Inflammatory Diseases

The UPS tightly regulates immune signaling pathways, and its dysregulation can lead to hyperactive inflammation or immunodeficiency. An example is the NF-κB pathway, which is regulated by ubiquitination [130]. A DUB A20 terminates NF-κB signaling by removing K63-ubiquitin chains and adding K48-chains to receptor-interacting protein 1 and other signaling proteins [131]. Loss-of-function mutations in the *A20* gene are linked to autoimmune and inflammatory conditions, such as lupus, rheumatoid arthritis, and psoriasis, because of the failure to properly shut off NF-κB and TNF receptor signaling [132].

In contrast, certain E3 ligases positively regulate immune signals. For example, TRAF6 is required for NF-κB activation downstream of Toll-like receptors. Mice with defective TRAF6 expression exhibit impaired inflammatory responses [133,134]. Defects in E3 ubiquitin ligase CBL-B cause hyperactive immune responses [135].

The UPS also plays a role in regulating immune checkpoints. DUBs, such as USP22, stabilize programmed death-ligand 1 in cancer cells and affect immune evasion [136]. Given the pervasive influence of the UPS on cytokine signaling, antigen presentation, and lymphocyte development, the system is a significant factor in immune-related diseases [137,138,139].

## 4. Emerging Therapeutic Technologies for Protein Homeostasis

The UPS fine-tunes the stability of key proteins, enabling cells to maintain their normal physiological functions. However, critical defects in this system can lead to diseases. This has motivated efforts to pharmacologically target specific components of the UPS. Unlike broad proteasome inhibitors that exert systemic effects, recent approaches have increasingly focused on directly targeting individual E3 ligases or DUBs [140,141].

### 4.1. Proteolysis-Targeting Chimeras (PROTACs): Hijacking the UPS for Targeted Degradation

PROTACs are heterobifunctional small molecules that enforce proximity between a target protein and an E3 ligase, most commonly cereblon (CRBN) or von Hippel–Lindau disease (VHL), thereby inducing ubiquitination and proteasomal degradation of target proteins [142]. Unlike classical occupancy-driven inhibitors, PROTACs can eliminate proteins that lack enzymatically active sites, enabling pharmacological access to “undruggable” targets. Clinically, this modality has matured into a late-stage development in oncology. For example, the estrogen receptor PROTAC vepdegestrant (ARV-471) is being evaluated in the phase III VERITAC-2 trial in estrogen receptor^+^/human epidermal growth factor receptor 2^−^ advanced/metastatic breast cancer [143] (Table 3).

### 4.2. Deubiquitinase-Targeting Chimeras (DUBTACs): Targeted Protein Stabilization by Deubiquitination

In contrast to targeted degradation, DUBTACs are designed to stabilize proteins by recruiting DUBs to remove ubiquitin chains from selected substrates, thereby opposing proteasomal commitment. This approach is attractive for restoring the levels of protective factors, such as tumor suppressors, or for rescuing destabilized folding mutants (e.g., cystic fibrosis transmembrane conductance regulator [CFTR] variants), where degradation is a major component of the loss-of-function [144].

Notably, DUBTAC expanded beyond the initial OTUB1-recruiting design [141]. More recently, USP7-based DUBTACs have been reported, supporting the generalizability of DUB recruitment chemistry and enabling the stabilization of disease-relevant substrates (including CFTR variants), as well as pathway-level modulation (e.g., AMP-activated protein kinase signaling) depending on the recruiter design and substrate context [145]. Collectively, DUBTACs establish a complementary therapeutic paradigm for PROTACs. Rather than eliminating pathogenic proteins [146], they aim to reinstate deficient protein function by preventing inappropriate turnover.

## 5. Conclusions

The UPS has transcended its historical definition as a waste management pathway and has become recognized as a command center for cellular life. From the precise timing of the cell cycle to the rapid mobilization of immune defenses, the UPS has ubiquitous functions and names. Across the core enzyme classes reviewed here, a recurring principle is that enzyme architecture dictates biological output. E1 enzymes provide ATP-driven activation and gatekeeping of the ubiquitin pool; E2 enzymes shape ubiquitin transfer chemistry and chain topology; E3 ligases impose substrate selectivity and pathway logic through degron recognition and regulated assembly; and DUBs confer reversibility and proofreading by trimming, remodeling, or erasing ubiquitin signals [147]. Together, these modules enable ubiquitin to function not simply as a degradation tag, but as a dynamic signaling language that governs cell cycle progression, DNA damage repair, immunity and inflammation, stress adaptation, and autophagy [47].

Therapeutically, the field is undergoing a clear transition from broad system-wide UPS perturbation to precise control of ubiquitin dynamics. Although proteasome inhibitors have validated the UPS as a druggable axis, their systemic liabilities motivate strategies that act at a higher resolution [148,149]. Direct small-molecule modulation of individual E3 enzymes or DUBs represents one such route. In parallel, proximity-inducing modalities have opened a new frontier in which PROTACs exploit endogenous E3 ligases, such as CRBN or VHL, to enforce target ubiquitination and degradation, enabling pharmacologic elimination of proteins previously considered “undruggable”. Several programs have advanced into late-stage clinical development [143]. Conversely, DUBTACs introduce a complementary paradigm of targeted protein stabilization by recruiting DUB activity to remove ubiquitin chains and prevent premature turnover, offering conceptual solutions for diseases driven by loss of function due to excessive degradation or folding instability [150].

Several priorities have emerged. First, mechanistic depth matters. Achieving therapeutic selectivity requires understanding which enzyme–substrate pairs causally drive a phenotype in a given cellular and tissue context. Second, the next generation of UPS-directed therapies will benefit from integrating degradation and stabilization strategies, enabling bidirectional control of protein abundance, as dictated by disease biology. Collectively, the progress summarized in this review supports the optimistic conclusion that by decoding and precisely manipulating the enzymatic logic of ubiquitination and deubiquitination, the UPS can be transformed from a foundational homeostatic system into a powerful and increasingly programmable therapeutic platform.

## Figures and Tables

**Table 1:** Classification and functional diversity of ubiquitination enzymes.

Enzyme Class		Mechanism of Transfer	Structural Feature	Proteins	Function
E1		Adenylation → thioester (ATP-dependent)	Adenylation domain Catalytic Cys domain	UBE1, UBE1L2	Initiates the cascade
E2		Transthioesterification (E1 → E2)	UBC domain (Catalytic Cys)	UBE2R1, UBE2N	Contributes to ubiquitin chain topology
E3	RING U-box	Scaffold (E2 → substrate)	Zinc (Zn^2+^)-coordinated RING finger domain	MDM2, c-CBL, SCF complex, APC/C complex,	Major determinant of substrate specificity
			U-box domain (H-bonds/Salt bridges, No Zn^2+^)	CHIP, UBE4B	Protein quality control (chaperone-linked)
	HECT	Thioester intermediate (E2 → E3 → substrate)	HECT domain (Catalytic Cys)	E6-AP, NEDD4, Smurf1/2	Ubiquitin chain type specificity (e.g., K63), Membrane trafficking
	RBR	RING-HECT Hybrid	RING1-IBR-RING2 (Catalytic Cys)	Parkin, HOIP, ARIH1	Mitophagy (Parkin), Linear chain assembly (LUBAC)

MDM2; Mouse double minute 2 homolog; c-CBL: Casitas B-lineage lymphoma; APC/C: anaphase-promoting complex/cyclosome; NEDD4: Neural precursor cell expressed, developmentally down-regulated protein 4; Smurf1/2: Smad ubiquitin regulatory factor 1; HOIP: HOIL-1-interacting protein; ARIH1: Ariadne RBR E3 ubiquitin protein Ligase 1; CHIP: Carboxy-terminus of Hsc70-interacting protein; UBE4B: Ubiquitination factor E4B.

**Table 2:** Key DUBs and specificities.

Family	Catalytic Type	Specificity	Proteins	Function
USP	Cys	Broad/promiscuous	CYLD, DUB3, USP1–8/9X/9Y/10–16/17L1/18–22/24–26/27X/28–54/L1	Protein stability and signaling regulation
UCH	Cys	Mainly mono-ubiquitin Small adducts	UCH-L1/3/5, BAP1	Ubiquitin recycling Chromatin regulation
OTU	Cys	Linkage specific (K48, K63, Linear)	A20, OTUB1/2, OTUD1/3/4/5/6A/6B/7A/7B, OTULIN, OTULINL, TRABID, VCPIP1, YOD1	Immune signaling regulation
MJD/Josephin	Cys	Prefers K63 chains	Ataxin-3/3L, JOSD1/2	Protein quality control
MINDY	Cys	K48-linked chains (Long chains preference)	MINDY1–4/4B	Molecular ruler
ZUFSP	Cys	K63-linked chains(Long chains preference)	ZUP1	DNA damage response
JAMM/MPN+	Zn^2+^	K63	AMSH/-LP, BRCC36, CSN5/6, EIF3F/H, MPND, MYSM1, PRPF8, RPN8/11	Protein stability, endosomal sorting regulation

CYLD: Cylindromatosis lysine 63 deubiquitinase; BAP1: BRCA1 associated protein 1; OTUB1: OTU domain-containing ubiquitin aldehyde-binding protein 1; OTUD1: OTU deubiquitinase 1; OTULIN: OTU deubiquitinase with linear linkage specificity; TRABID: TRAF-binding domain-containing protein; VCPIP1: Valosin-containing protein (VCP/p97) interacting protein 1; YOD1: Ubiquitin thioesterase OTU1; JOSD1: Josephin domain-containing protein 1; ZUP1: Zinc finger-containing ubiquitin peptidase 1; AMSH: associated with the SH3 domain of STAM; BRCC36: Lys-63-specific deubiquitinase BRCC36; CSN5: COP9 signalosome subunit 5; EIF3F: Eukaryotic translation initiation factor 3 subunit F; MPND: Mpr1/Pad1 N-terminal domain-containing protein; MYSM1: Myb-like, SWIRM, and MPN domains 1; PRPF8: Pre-mRNA-processing-splicing factor 8.

**Table 3:** PROTACs in clinical trials.

PROTAC	E3 Ligase	Target	Indications	Phase	NCT Number
ARV-110	CRBN	AR	Prostate cancer	I/II	NCT03888612
ARV-471	CRBN	ER	Breast cancer	III	NCT05654623
ARV-766	VHL	AR	Prostate cancer	I/II	NCT05067140
BGB-16673	CRBN	BTK	B-cell malignancies	I/II	NCT06973187
BMS-986458	CRBN	BCL6	Non-Hodgkin lymphoma	I/II	NCT06090539
BTX-9341	CRBN	CDK4/6	Breast cancer	I	NCT06515470
CC-94676	CRBN	AR	Prostate cancer	III	NCT06764485
CFT8634	CRBN	BRD9	Synovial sarcoma and SMARCB1-loss tumors	I/II	NCT05355753
CFT8919	CRBN	EGFR	Non-small cell lung cancer	I	NCT06641609
DT2216	VHL	BCL-xL	Liquid and Solid tumors	I/II	NCT04886622
FHD-609	CRBN	BRD9	Synovial sarcoma	I	NCT04965753
GT20029	VHL	AR	Androgenetic alopecia and acne vulgaris	II	NCT06692465
KT-474	CRBN	IRAK4	Autoimmune diseases	II	NCT06058156
NX-5948	CRBN	BTK	B-cell malignancies	I	NCT05131022

AR: Androgen receptor; ER: Estrogen receptor; BTK: Bruton’s tyrosine kinase; BCL6: B-cell lymphoma 6; CDK: Cyclin-dependent kinase; BRD9: Bromodomain-containing protein 9; EGFR: Epidermal growth factor receptor; BCL-xL: B-cell lymphoma—extra-large; IRAK4: Interleukin-1 receptor-associated kinase 4; SMARCB1: SWI/SNF related, matrix associated, actin dependent regulator of chromatin, subfamily B, member 1.

## Data Availability

No new data were created or analyzed in this study. Data sharing is not applicable to this article.

## Abbreviations

The following abbreviations are used in this manuscript:

AD	Alzheimer’s disease
AMSH	Associated with the SH3 domain of STAM
ATP	Adenosine triphosphate
BAP1	BRCA1-associated protein 1
BRCA1	Breast cancer type 1 susceptibility protein
BRCC36	Lys-63-specific deubiquitinase BRCC36
CBL	Casitas B-lineage lymphoma
CFTR	Cystic fibrosis transmembrane conductance regulator
CHIP	Carboxy-terminus of Hsc70-interacting protein
CRBN	Cereblon
CSN5	COP9 signalosome subunit 5
CYLD	Cylindromatosis lysine 63 deubiquitinase
Cys	Cysteine
DUBs	Deubiquitinases
DUBTACs	Deubiquitinase-targeting chimeras
E1	E1 ubiquitin-activating enzyme
E2	E2 ubiquitin-conjugating enzyme
E3	E3 ubiquitin ligase
DNA	Deoxyribonucleic acid
DUBs	Deubiquitinating enzymes
E6-AP	E6-associated protein
FAT10	Human leukocyte antigen-F adjacent transcript 10
FBXW7	F-Box and WD repeat domain containing 7
Gly	Glycine
Hsp	Heat shock protein
HECT	Homologous to E6-AP C-Terminus
HOIL-1	Heme-oxidized IRP2 ubiquitin ligase-1
HOIP	HOIL-1-interacting protein
JAMM/MPN^+^	Jun activation domain-binding protein 1/Mpr1-Pad1-N-terminal/MOV34 metalloenzyme
JOSD1	Josephin domain-containing protein 1
Lys	Lysine
LUBAC	Linear ubiquitin chain assembly complex
MDM2	Mouse double minute 2 homolog
MJD	Machado–Joseph disease proteases
NEDD4	Neural precursor cell expressed, developmentally down-regulated protein 4
MINDY	Motif interacting with ubiquitin-containing novel DUB family
NF-κB	Nuclear factor kappa-light-chain-enhancer of activated B cells
OTU	Ovarian tumor protease
OTUB1	OTU domain-containing ubiquitin aldehyde-binding protein 1
OTUD1	OTU deubiquitinase 1
OTULIN	OTU deubiquitinase with linear linkage specificity
PD	Parkinson’s disease
PINK1	PTEN-induced kinase 1
PROTACs	Proteolysis-targeting chimeras
RBR	RING-Between-RING
RING	Really interesting new gene
RPN11	Regulatory particle non-ATPase 11
SCF	SKP1-CUL1-F-box protein
SUMO	Small ubiquitin-like modifier
TNF	Tumor necrosis factor
TRAF6	TNF receptor-associated factor 6
UBA1	Ubiquitin-like modifier activating enzyme 1
UBA6	Ubiquitin-like modifier activating enzyme 6
UBC	Ubiquitin-conjugating
UBE1	Ubiquitin-activating enzyme E1
UBE1L2	UBE1-like protein 2
UBE2	Ubiquitin-conjugating enzyme E2
UBL	Ubiquitin-like
UCH	Ubiquitin carboxy-terminal hydrolase
UPS	Ubiquitin–proteasome system
USP	Ubiquitin specific protease
VHL	Von Hippel–Lindau disease
WW	Tryptophan–tryptophan
ZUFSP	Zinc finger-containing ubiquitin peptidase
